# *Clostridioides difficile* in COVID-19 Patients, Detroit, Michigan, USA, March–April 2020

**DOI:** 10.3201/eid2609.202126

**Published:** 2020-09

**Authors:** Avnish Sandhu, Glenn Tillotson, Jordan Polistico, Hossein Salimnia, Mara Cranis, Judy Moshos, Lori Cullen, Lavina Jabbo, Lawrence Diebel, Teena Chopra

**Affiliations:** Detroit Medical Center, Detroit, Michigan, USA (A. Sandhu, J. Polistico, H. Salimnia, M. Cranis, J. Moshos, L. Cullen, L. Jabbo, T. Chopra);; Wayne State University School of Medicine, Detroit (A. Sandhu, J. Polistico, H. Salimnia, L. Diebel, T. Chopra);; GST Micro LLC, Henrico, Virginia, USA (G. Tillotson)

**Keywords:** *Clostridiodes difficile*, bacteria, bacterial infections, co-infection, diarrhea, COVID-19, coronavirus disease, SARS-CoV-2, severe acute respiratory syndrome coronavirus 2, viruses, respiratory infections, zoonoses, Detroit, Michigan, United States, antibiotics, antimicrobial drug resistance, nosocomial infections

## Abstract

We describe 9 patients at a medical center in Detroit, Michigan, USA, with severe acute respiratory syndrome coronavirus 2 and *Clostridioides difficile*. Both infections can manifest as digestive symptoms and merit screening when assessing patients with diarrhea during the coronavirus disease pandemic. These co-infections also highlight the continued importance of antimicrobial stewardship.

Coronavirus disease (COVID-19), which is caused by infection with severe acute respiratory syndrome coronavirus 2 (SARS-CoV-2), predominantly includes pulmonary symptoms; however, <10% of cases also include gastrointestinal events, including abdominal pain, diarrhea, and vomiting (*1*–*4*). During the COVID-19 pandemic, clinicians must be vigilant of co-infections in patients with COVID-19.

Several studies have collected data on concomitant antibiotic use in patients with COVID-19. A single-center study of 52 critically ill patients cited hospital-acquired infection in only 7 (13.5%) patients, yet 49 (94%) patients received antibiotic therapy (*5*). Another study, which analyzed 113 deceased patients from a cohort of 799 moderate-to-severely ill COVID-19 patients during January 13–February 12, 2020, reported that 105 (93%) deceased patients and 144 (89%) survivors had received empiric antibacterial therapy with either moxifloxacin, cefoperazone, or azithromycin (*6*). These antibiotics are strongly associated with *Clostridioides difficile* infection (CDI) (*7*). We report an observation of CDI as a co-occurrence or sequalae of overuse of antibiotics in COVID-19 patients.

We conducted a clinical surveillance review of CDI for all laboratory-confirmed COVID-19 patients treated at any of the hospitals belonging to Detroit Medical Center (Detroit, Michigan, USA). We screened patients by using TheraDoc software (https://www.theradoc.com) during March 11–April 22, 2020. We abstracted data regarding baseline demographics, medical history, symptoms, laboratory values, microbiologic findings, concomitant antibiotic use, and treatment for CDI. We obtained institutional review board approval for this study.

We identified 9 cases of co-infection with SARS-CoV-2 and *C. difficile*. This cohort mainly included elderly patients who were predominantly female (Table). The rate of CDI at the center was 3.32/10,000 patient-days during January–February 2020 and increased to 3.6/10,000 patient-days during March–April 2020.

**Table:** Baseline demographic and clinical characteristics of 9 patients with *Clostridioides difficile* and severe acute respiratory syndrome coronavirus 2 co-infection, Detroit, Michigan, USA, March–April 2020*

Characteristic	Value
Age, y, median	75
Sex	
F	7
M	2
Race	
African American	6
Caucasian	1
Unknown	2
Hospitalization in prior 60 d	5
Required intensive care unit and vasopressors	4
ATLAS score, median†	6
Charlson comorbidity index score, median	8
Symptoms at admission	
Cough	4
Shortness of breath	3
Fever	4
Diarrhea and abdominal pain	2
Laboratory results	
Ferritin, ng/mL, median‡	1,459.4
Leukocyte count, x 10^3^ cells/mm^3^), average	12.0
Creatinine, mg/dL, average§	4.22
Microbiologic findings	
Blood culture positive	2
Respiratory culture positive	2

*Values indicate no. patients unless otherwise indicated. Some patients had >1 symptom. †Scoring information available at https://www.mdcalc.com/atlas-score-clostridium-difficile-infection. ‡Ferritin was only obtained in 8 patients. §Three patients were on dialysis.

We noted prior CDI in 3 patients; these infections occurred 1–4 months before admission. All patients were confirmed to be positive for *C. difficile* by PCR and showed symptoms of diarrhea in addition to other characteristic signs and symptoms, such as abdominal pain, nausea, and vomiting. Two patients had diarrhea and were found to be positive for *C. difficile* at admission, whereas the remaining 7 had onset of diarrhea only after COVID-19 diagnosis; median duration from CDI diagnosis to COVID-19 diagnosis in these 7 patients was 6 days. This group of patients were severely ill, having high ATLAS scores (https://www.mdcalc.com/atlas-score-clostridium-difficile-infection) and multiple underlying conditions; hypertension (n = 8) and diabetes (n = 5) were the most frequent of these conditions.

Three patients received antibiotics in the month before admission; 8 received antibiotics at admission. One patient was initiated on antibiotics on day 15; this patient was also receiving antibiotics the month before admission. The most commonly administered antibiotics were cefepime (n = 5), ceftriaxone (n = 3), meropenem (n = 2), and azithromycin (n = 2). Specific CDI therapies were oral vancomycin (n = 6); vancomycin and intravenous metronidazole (n = 1); no treatment (n = 1); and a combination of oral vancomycin, intravenous metronidazole, rectal vancomycin, fidaxomicin, and fecal microbiota transplantation (n = 1). One patient who did not receive antibiotics was considered to be colonized with *C. difficile*. Four (44.4%) patients died during hospital admission, 1 (11.1%) was discharged to hospice, 1 (11.1%) is still hospitalized, and 3 (33.3%) were discharged to a long-term care facility.

CDI is a challenging disease, with a recurrence rate of 15%–20% and a mortality rate of 5% (*8*). When CDI is present as a co-infection with COVID-19, CDI therapy can be difficult to monitor if diarrhea persists because of COVID-19.

These cases highlight the importance of judicious use of antibiotics for potential secondary bacterial infection in patients with COVID-19. Antibiotics are known to have unintended consequences, such as *C. difficile* infection. All 9 patients received antibiotics; the median duration of antibiotic use before PCR-positive CDI was 5 days. All patients in our cohort were elderly, an age group at higher risk for complications from overuse of antibiotics, such as adverse events, antibiotic resistance, and concomitant infections like CDI (*9*). Secondary infections on top of CDI can increase the risk for death in patients with severe COVID-19; in this cohort, 4 patients died and 1 was discharged to hospice. To prevent CDI co-infections during the COVID-19 pandemic, integrated use of antimicrobial stewardship is needed to monitor appropriate antibiotic use.

Symptoms of CDI can complicate diagnosis of COVID-19 because both conditions can have similar manifestations; in a study of 206 COVID-19 patients, 19.4% had diarrhea as the first symptom onset (*10*). Of the 2 patients who had CDI diagnosed at admission, 1 patient solely had gastrointestinal symptoms, which possibly led to delayed diagnosis of COVID-19. Both COVID-19 and CDI should be considered when evaluating patients with diarrhea during the COVID-19 pandemic. Distinguishing between actual CDI versus colonization also is vital; 1 patient in our cohort was colonized. A limitation of this study is the small number of cases. However, in the face of the COVID-19 pandemic and the extensive use of antibiotics, clinicians should remain aware of possible CDI and SARS-CoV-2 co-infection. 

## References

[1] US Centers for Disease Control and Prevention. Interim clinical guidance for management of patients with confirmed coronavirus disease (COVID-19) [cited 2020 May 1]. https://www.cdc.gov/coronavirus/2019-ncov/hcp/clinical-guidance-management

[2] Guan WJ, Ni ZY, Hu Y, Liang WH, Ou CQ, He JX, et al.; China Medical Treatment Expert Group for Covid-19. Clinical Characteristics of Coronavirus Disease 2019 in China. N Engl J Med. 2020;382:1708–20. 10.1056/NEJMoa2002032 32109013 PMC7092819

[3] Chen N, Zhou M, Dong X, Qu J, Gong F, Han Y, et al. Epidemiological and clinical characteristics of 99 cases of 2019 novel coronavirus pneumonia in Wuhan, China: a descriptive study. Lancet. 2020;395:507–13. 10.1016/S0140-6736(20)30211-7 32007143 PMC7135076

[4] Wang D, Hu B, Hu C, Zhu F, Liu X, Zhang J, et al. Clinical characteristics of 138 hospitalized patients with 2019 novel coronavirus-infected pneumonia in Wuhan, China. JAMA. 2020;323:1061–9. 10.1001/jama.2020.1585 32031570 PMC7042881

[5] Yang X, Yu Y, Xu J, Shu H, Xia J, Liu H, et al. Clinical course and outcomes of critically ill patients with SARS-CoV-2 pneumonia in Wuhan, China: a single-centered, retrospective, observational study. Lancet Respir Med. 2020;8:475–81. 10.1016/S2213-2600(20)30079-5 32105632 PMC7102538

[6] Chen T, Wu D, Chen H, Yan W, Yang D, Chen G, et al. Clinical characteristics of 113 deceased patients with coronavirus disease 2019: retrospective study. BMJ. 2020;368:m1091. 10.1136/bmj.m1091 32217556 PMC7190011

[7] Brown KA, Khanafer N, Daneman N, Fisman DN. Meta-analysis of antibiotics and the risk of community-associated *Clostridium difficile* infection. Antimicrob Agents Chemother. 2013;57:2326–32. 10.1128/AAC.02176-12 23478961 PMC3632900

[8] Guh AY, Mu Y, Winston LG, Johnston H, Olson D, Farley MM, et al. Emerging Infections Program *Clostridioides difficile* Infection Working Group. Trends in US burden of *Clostridium difficile* infection and outcomes. N Engl J Med. 2020;382:1320–30. 10.1056/NEJMoa1910215 32242357 PMC7861882

[9] Biedron C, Chopra T. Issues surrounding antibiotic use in older adults. Curr Transl Geriatr Exp Gerontol Rep. 2013;2:151–8. 10.1007/s13670-013-0050-9

[10] Han C, Duan C, Zhang S, Spiegel B, Shi H, Wang W, et al. Digestive symptoms in COVID-19 patients with mild disease severity: clinical presentation, stool viral RNA testing, and outcomes. Am J Gastroenterol. 2020;1; Epub ahead of print. 10.14309/ajg.0000000000000664 32301761 PMC7172493

